# Plasma endothelial cells-derived extracellular vesicles promote wound healing in diabetes through YAP and the PI3K/Akt/mTOR pathway

**DOI:** 10.18632/aging.103366

**Published:** 2020-06-22

**Authors:** Feng Wei, Aixue Wang, Qing Wang, Wenrui Han, Rong Rong, Lijuan Wang, Sijia Liu, Yimeng Zhang, Chao Dong, Yanling Li

**Affiliations:** 1 Department of Dermatology, The Second Hospital of Hebei Medical University, Shijiazhuang 050000, Hebei, P.R. China

**Keywords:** skin wound healing, diabetes, plasma endothelial cells-derived extracellular vesicle, senescence, YAP

## Abstract

Extracellular vesicles are involved in skin wound healing and diabetes. After enrichment and identification, plasma endothelial cells-derived-extracellular vesicles were cocultured with skin fibroblasts or HaCaT. The gain-and loss-of functions were performed to measure fibroblast proliferation, senescence, and reactive oxygen species. Levels of senescence-related proteins, senescence-associated secretory phenotypes, vascular markers, YAP and the PI3K/Akt/mTOR pathway-related proteins were determined. Diabetic mice were induced to establish skin wound model. After endothelial cells-derived-extracellular vesicles were injected into skin wound modeling mice, skin wound healing was evaluated. Endothelial cells-derived-extracellular vesicles treatment enhanced fibroblast proliferation, and decreased senescence through the elevation of YAP nuclear translocation and activation the PI3K/Akt/mTOR pathway. YAP inhibition reversed the effect of plasma endothelial cells-derived-extracellular vesicles on fibroblast proliferation. Endothelial cells-derived-extracellular vesicles also promoted wound healing in diabetic mice, increased microvascular density, collagen deposition, macrophage infiltration and positive rates of vascular markers, and inhibited YAP phosphorylation and senescence. Plasma endothelial cells-derived-extracellular vesicles prevent fibroblast senescence and accelerate skin wound healing in diabetic mice by reducing YAP phosphorylation and activating the PI3K/Akt/mTOR pathway. This study may provide novel insights for skin disorders in diabetic mice.

## INTRODUCTION

Skin disorders are found in nearly one third of all diabetic populations and always occur before diagnosis, playing a considerable role in the preliminary recognition of diabetes [1]. Diabetic dermopathy is the most frequent skin lesion in diabetic patients, accounting for 7% to 70% of diabetic patients [2]. Diabetic wounds impacted by insufficient angiogenesis, often result in non-healing ulcers, and decreased vascularity and capillary density; and owing to the high risk of chronic wound infection, they often end up with amputations [3, 4]. Moreover, 40%-70% of all lower extremity amputations are related to diabetes, of which 85% are preceded by diabetic foot ulcers [5]. The non-healing wounds are caused by a complex mechanism of deficient or improper immune response, poor arterial circulation and oxygenation, peripheral neuropathy, and wretched general health due to diabetes [4]. A literature review suggests that diabetes leads to skin wounds by modulating one or more biological processes of hemostasis, inflammation, proliferation and remodeling [6]. Particularly, chronic skin wounds feature with senescent fibroblasts, which lose their replication ability when preserving the metabolic functions [7]. In view of these points, we are encouraged to search for novel approaches to diabetic wound healing from the aspects of inflammation, senescence and proliferation of fibroblasts.

Extracellular vesicles (EVs) are a kind of membrane-bound vesicles released from cell surface, which have profound impacts by acting as mediators of intercellular communication on normal physiology and pathological processes [8]. EVs are thought to carry signals between cells during normal development and homeostasis, and they have also been implicated in neuronal functions, neurodegenerative disorders, cancers, immune responses, infections, and senescence [9]. A review notes encouraging results for administration of EVs in a wide range of clinical animal models of skin wounds [10]. Endothelial cell-derived EVs (ED-EVs) contain functional cell-surface proteins and other ones participating in endothelial cell activities, and maintain the abilities of endothelial cells to enhance monocyte adhesion, cell migration, and angiogenesis [11]. ED-EVs undertake intercellular communication during the development of atherosclerosis, coronary artery disease, and type 2 diabetes via the transfer of cellular contents [12]. Importantly, human endothelial progenitor cells-derived EVs are reported to accelerate diabetic wound healing by promoting angiogenesis through extracellular signal-regulated kinases 1 and 2 signaling pathway [13]. In light of these references, we attempted to evaluate the mechanism of ED-EVs in skin wound healing in diabetic mice from the perspective of fibroblast senescence.

## RESULTS

### Identification of plasma ED-EVs and skin fibroblasts

**Figure 1:**
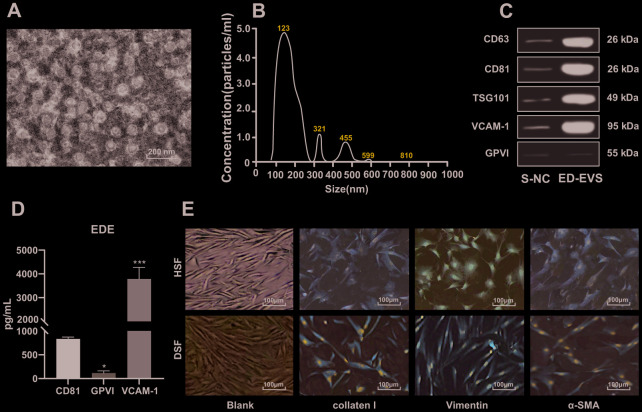
**Identification of plasma ED-EVs and skin fibroblasts.** (**A**) The morphology and size of EVs was observed by transmission electron microscope. (**B**) The nanoparticle tracking software showed that the average diameter of ED-EVs was 123 ± 8 nm, and the concentration was 5.2 × 10^7^ particles/mL. (**C**) Western blot analysis showed that CD63, CD81, TSG101 and VCAM-1 were higher in plasma ED-EVs than those in S-NC, and GPVI was markedly low in both groups. S-NC is the supernatant after immunoprecipitation. (**D**) The amount of proteins carried in ED-EVs was quantified by ELISA; compared with CD81, * *p* < 0.05, *** *p* < 0.001. (**E**) Collagen I and Vimentin were positive and α-SMA was weakly positive as immunocytochemistry indicated. Data in panel C were analyzed by two-way ANOVA, and in panel **D** were analyzed by one-way ANOVA, followed by Tukey's multiple comparisons test. Repetitions = 3.

After enrichment of ED-EVs, the morphology of EVs was observed under a transmission electron microscope (Figure 1A). The average diameter of ED-EVs was 123 ± 8 nm, and the concentration was 5.2 × 10^7^ particles/mL (Figure 1B). CD63, CD81, TSG101 and VCAM-1 were significantly higher in plasma ED-EVs than those in S-NC, and GPVI was markedly low in both groups (Figure 1C). The level of proteins carried in ED-EVs are presented in Figure 1D. Collagen I and Vimentin were positive and α-SMA was weakly positive in primary skin fibroblasts of diabetic patients and healthy volunteers (all *p* < 0.05) (Figure 1E).

### Plasma ED-EVs inhibit DSF premature senescence

**Figure 2:**
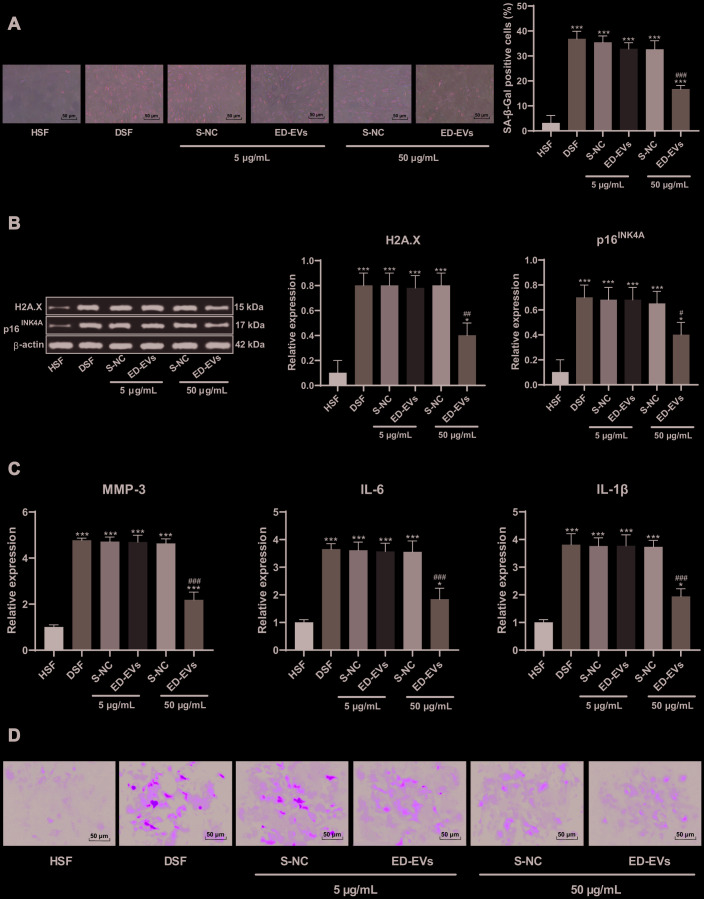
**Plasma ED-EVs inhibit DSF premature senescence.** (**A**) SA-β-gal detected skin fibroblast senescence after ED-EVs treatment at the concentration of 5 μg/mL or 50 μg/mL. At the same passage times (3 generations) and culture conditions, compared with the healthy volunteers' skin fibroblasts (HSF), the activity of SA-β-gal in skin fibroblasts of diabetic patients (DSF) was increased significantly, but decreased after the action of ED-EVs; (**B**) Western blot analysis detected the levels of H2A.X, p16^INK4A^ in fibroblast; (**C**) ELISA measured SASP level (MMP-3, IL-6 and IL-1β) in fibroblast; (**D**) H2DCFDA probe measured ROS level in in fibroblast. compared with the blank group, ***p < 0.001. Data were analyzed by one-way ANOVA, followed by Tukey's multiple comparisons test. Repetitions = 3.

DSF showed signs of early aging at the same passage times (the third generation) and culture conditions (Figure 2A–2E). S-NC and plasma ED-EVs were coincubated with DSF respectively, and their effects on cell senescence were detected by SA-β-gal assay (Figure 2A). At the concentration of 5 μg/mL, there were no significant differences between S-NC and plasma ED-EVs, neither of which significantly inhibited cell senescence. However, at the concentration of 50 μg/mL, plasma ED-EVs significantly inhibited the senescence of DSF, while S-NC could not. The expression of H2A. X and p16^INK4a^ was decreased (Figure 2B), and the levels of SASP-related proteins (MMP-3, IL-6, IL-1β) were decreased significantly in DSF (Figure 2C) after 50 μg/mL ED-EVs treatment. In addition, 50 μg/mL ED-EVs significantly reducde ROS level in DSF (Figure 2D) (all *p* < 0.05).

### Plasma ED-EVs activate YAP nuclear translocation and promote DSF proliferation

**Figure 3:**
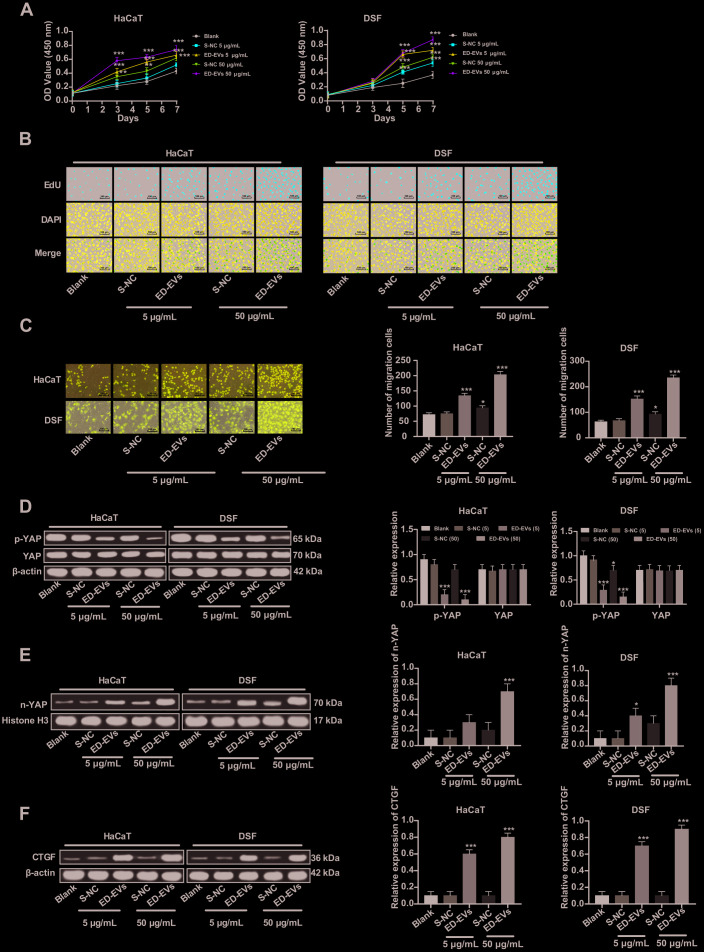
**Plasma ED-EVs activate YAP nuclear translocation and promote DSF proliferation.** (**A**) CCK-8 detected DSF viability after ED-EVs or S-NC treatment. (**B**) EdU assay measured DSF proliferation after ED-EVs or S-NC treatment. (**C**) Transwell assay measured fibroblast migration after ED-EVs or S-NC treatment. (**D**–**F**) Western blot analysis measured YAP phosphorylation in DSF, and YAP and CTGF expression in nucleus. Compared with the blank group, *p < 0.05, **p < 0.01, ***p < 0.001. Data in panels (**C**, **E** and **F**) were analyzed by one-way ANOVA, and data in panel **D** were analyzed by two-way ANOVA, followed by Tukey's multiple comparisons test. Repetitions = 3.

The effects of ED-EVs and S-NC at different concentrations on the proliferation of DSF and HaCaT cells are shown in Figure 3A, 3B. At almost all time points, DSF cocultured with ED-EVs were significantly better in viability and proliferation than those cocultured with S-NC, while there were no remarkable differences between 50 μg/mL ED-EVs and 5 μg/mL S-NC in HaCaT cells on the 7^th^ day. Compared with S-NC, plasma ED-EVs promoted the migration of DSF and HaCaT cells (Figure 3C). At the same total protein levels, the number of migrating DSF cocultured with plasma ED-EVs was significantly larger than those cocultured with S-NC (all *p* < 0.05).

YAP was dephosphorylated and transported to the nucleus (Figure 3D, 3E) after plasma ED-EVs treatment, and its downstream effector protein CTGF was activated (Figure 3F) (all *p* < 0.05), which can promote the proliferation of fibroblasts and collagen deposition product [14].

### Inhibition of YAP can reverse the effect of plasma ED-EVs on DSF proliferation

**Figure 4:**
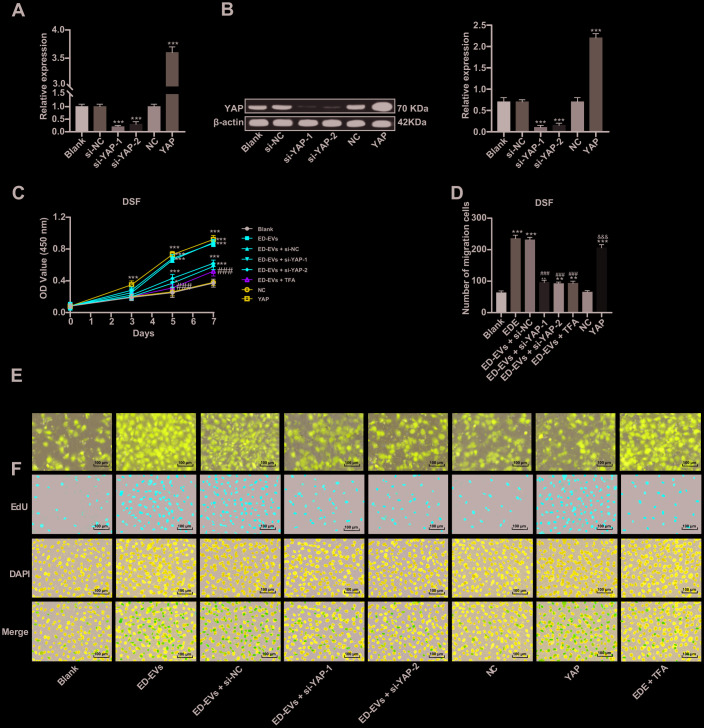
**Inhibition of YAP can reverse the effect of plasma ED-EVs on DSF proliferation.** (**A**, **B**) YAP expression in different transfected DSF was detected by RT-qPCR and western blot analysis. (**C**) CCK-8 detected DSF viability after different S-NC treatment. (**D**, **E**) Transwell assay measured DSF migration after different treatment. (**F**) Representative images of EdU results in different transfected DSF. Compared with the blank group, *p < 0.05, **p < 0.01, ***p < 0.001; compared to the ED-EVs group, ###*p* < 0.001; compared to the NC group, &&& *p* < 0.001. Data in panels (**A**, **B** and **D**) were analyzed by one-way ANOVA, and data in panel **C** were analyzed by two-way ANOVA, followed by Tukey's multiple comparisons test. Repetitions = 3.

Two pieces of si-YAP were transfected into DSF to downregulate YAP expression (Figure 4A, 4B). In DSF with downregulated YAP, the promoting effect of plasma ED-EVs on DSF proliferation and migration was inhibited (Figure 4C–4F). While DSF with overexpressing YAP showed similar effects to those with plasma ED-EVs treatment, with significant increases in cell proliferation and migration (Figure 4C–4F). The YAP inhibitor super TDU TFA (TFA) was transfected into plasma ED-EVs-treated DSF, which could inhibit the interaction between YAP and TEAD [15]. After YAP inhibition, the effect of plasma ED-EVs on DSF proliferation and migration was reversed (Figure 4C–4F) (all *p* < 0.05).

### Plasma ED-EVs inhibit DSF premature senescence through the PI3K/Akt/mTOR pathway

**Figure 5:**
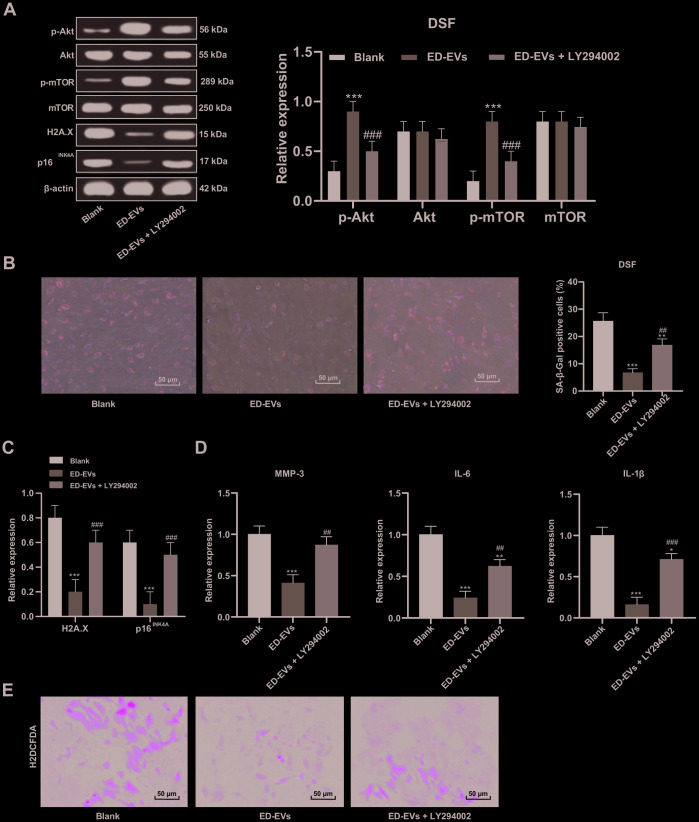
**Plasma ED-EVs inhibit DSF premature senescence through the PI3K/Akt/mTOR pathway.** (**A** and **C**) Western blot analysis detected levels of PI3K/Akt/mTOR pathway-and senescence-related proteins. (**B**) SA-β-gal assay detected DSF senescence. (**D**) ELISA measured SASP level (MMP-3, IL-6 and IL-1β) in DSF. (**E**) H2DCFDA probe measured ROS level in DSF. Compared with the blank group, *p < 0.05, **p < 0.01, ***p < 0.001; compared to the ED-EVs group, #*p* < 0.05, ##*p* < 0.01, ###*p* < 0.001. Data in panels (**A** and **D**) were analyzed by two-way ANOVA, and data in panels (**B**, **C** and **E**) were analyzed by one-way ANOVA, followed by Tukey's multiple comparisons test. Repetitions = 3.

As the PI3K/Akt/mTOR pathway is a known regulator of endothelial senescence [16], we tested the involvement of pathway members in DSF. The phosphorylation of Akt and mTOR in DSF was significantly upregulated after ED-EVs treatment (Figure 5A). LY294002 is a PI3K/Akt pathway inhibitor. When LY294002 was combined with ED-EVs, Akt and mTOR phosphorylation in DSF was decreased (Figure 5A). LY294002 reversed the inhibitory effects of plasma ED-EVs on DSF senescence, presenting with reduced levels of H2A.X, p16^INK4a^, SASP (MMP-3, IL-6, IL-1 β) (Figure 5B–5D), and increased ROS level (Figure 5E) (all *p* < 0.05).

### Plasma ED-EVs promote skin wound healing in diabetic mice

**Figure 6:**
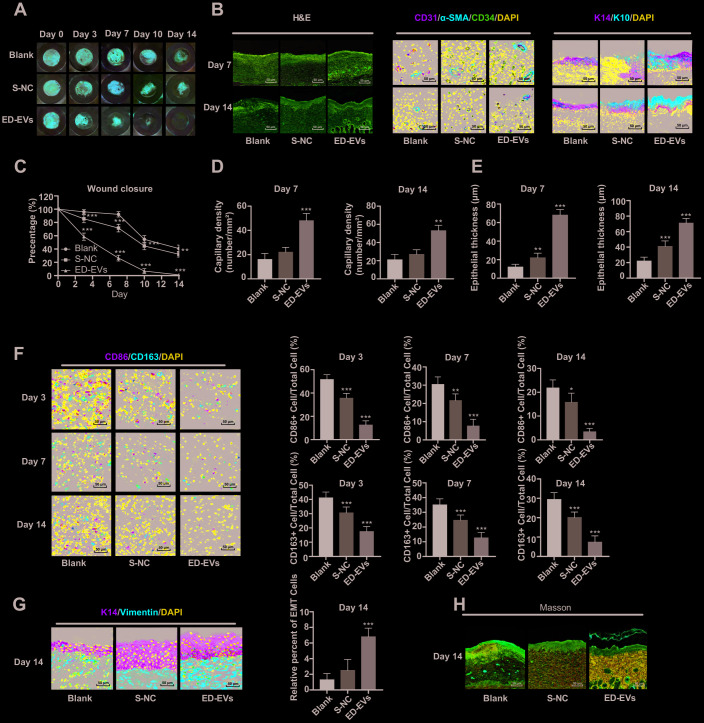
**Plasma ED-EVs promote skin wound healing in diabetic mice.** (**A** and **C**) The wound area of diabetic mice was measured on the 0, 3, 7, 10 and 14 days after skin wound modeling (n = 6). On the 3^rd^, 7^th^ and 14^th^ day after skin wound modeling, the pathological changes (**B, H**), vascular markers (**B**, **D**), re-epithelialization (**E**), macrophage infiltration and EMT (**F**, **G**) and collagen deposition (**F**) in the skin of diabetic mice were detected by HE staining (n = 3), immunofluorescence (n = 6) and Masson staining (n = 3). Compared with the blank group, *p < 0.05, **p < 0.01, ***p < 0.001. Data in panel (**C**) were analyzed by two-way ANOVA, and data in panels (**D**–**G**) were analyzed by one-way ANOVA, followed by Tukey's multiple comparisons test. Repetitions = 3.

No adverse reaction was observed in the process of skin wound model experiment in diabetic mice. As is shown in Figure 6A and 6C, the wounds in mice of all groups contracted with time, but the wound size of ED-EVs-treated mice was smaller than that in the control group. Of especial note, the wounds of ED-EVs-treated mice were almost healed on the 14^th^ day, while the wounds of control mice were not healed at all. ED-EVs significantly improved the early microvascular density of wound tissues (Figure 6B). The number of CD34- and CD31-positive cells in ED-EVs-treated mice were much more than those in the other mice (Figure 6B, 6D). ED-EVs promoted the re-epithelialization of the diabetic wound earlier and increase its epidermal maturity (Figure 6B, 6E), and effectively inhibited the inflammatory response of diabetic wound, increased the proportion of type II macrophages (CD163^+^)/type I macrophages (CD86^+^) (Figure 6F), and presented with better epithelial-mesenchymal transition (EMT) (Figure 6G), and more collagen deposition in ED-EVs-treated mice (Figure 6H) (all *p* < 0.05).

### Plasma ED-EVs inhibit premature senescence of skin fibroblasts in diabetic mice through the PI3K/Akt/mTOR pathway and YAP nuclear translocation

**Figure 7:**
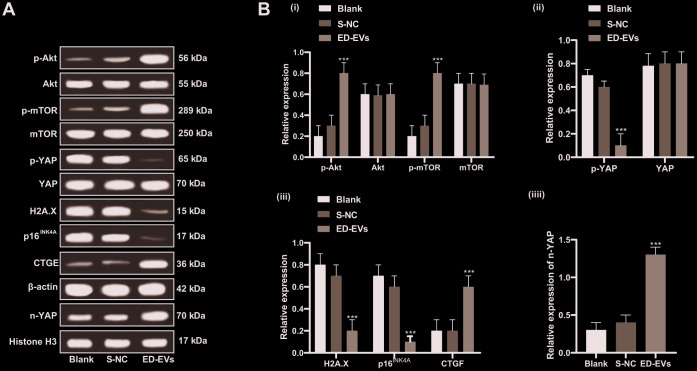
**Plasma ED-EVs inhibit premature senescence of skin fibroblasts in diabetic mice through the PI3K/Akt/mTOR pathway and YAP nuclear translocation.** (**A**, **B**) Western blot analysis detected levels of PI3K/Akt/mTOR pathway-and senescence-related proteins, YAP phosphorylation in fibroblasts, and YAP and CTGF expression in nucleus. Compared with the blank group, ***p < 0.001. Histone H3 was used as the internal parameter of YAP expression in nucleus and β-actin as the internal parameter of cytoplasmic proteins. Data in panel **B** (i, ii, iii) were analyzed by two-way ANOVA, and data in panel **B** (iv) were analyzed by one-way ANOVA, followed by Tukey's multiple comparisons test. n = 5.

Under the treatment of plasma ED-EVs, the PI3K/Akt/mTOR pathway was activated in the skin tissue of diabetic mice, YAP phosphorylation was significantly reduced, YAP level in the nucleus was increased, the expression of H2A. X, p16^INK4a^ was significantly downregulated, and CTGF level was significantly increased (all *p* < 0.05; Figure 7A, 7B). In summary, plasma ED-EVs could inhibit fibroblast senescence in diabetic mice by activating PI3K/Akt/mTOR pathway and promoting YAP nuclear translocation.

## DISCUSSION

**Figure 8:**
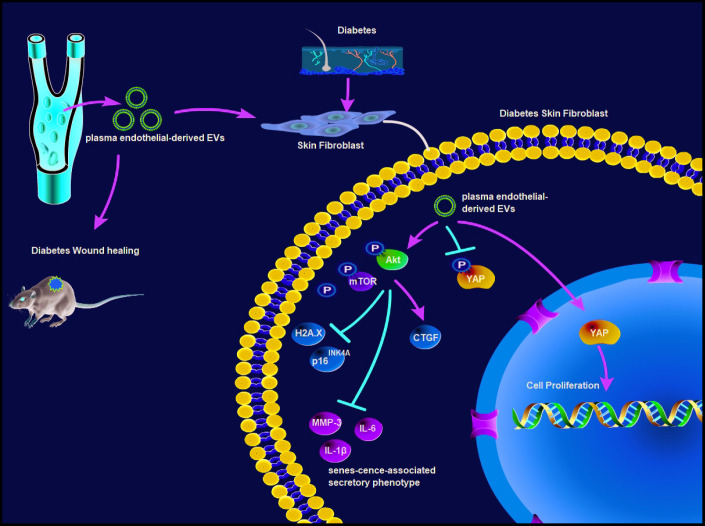
**Plasma ED-EVs prevent fibroblast senescence and accelerate skin wound healing in diabetic mice by promoting YAP nuclear translocation and activating the PI3K/Akt/mTOR pathway.**

Chronic wound in diabetes is a pivotal clinical issue, and the incidence is expected to elevate owing to an increased prevalence of diabetes [4]. EVs are documented to promote tissue repair and regeneration in animal models of wound healing, cardiac ischemia, diabetes and lung fibrosis [17]. It triggered us to figure out ED-EVs-based therapeutics for diabetic wound healing. Collectively, we unveiled a novel mechanism that plasma ED-EVs could prevent fibroblast senescence and accelerate skin wound healing in diabetic mice by downregulating YAP phosphorylation and activating the PI3K/Akt/mTOR pathway (Figure 8).

ED-EVs treatment increased proliferation and migration of fibroblasts, and decreased fibroblast senescence and levels of ROS, H2A.X, p16^INK4a^ and SASP (MMP-3, IL-6 and IL-1β). Due to the secretion of SASP-related proteins (including pro-inflammatory cytokines, chemokines, growth factors and proteases), senescent cells can promote chronic inflammation and change the microenvironment of tissues and functions of adjacent cells, leading to increased susceptibility for skin disorders [18, 19]. The senescence progression is accompanied with enhanced ROS level and activation of histone H2A.X [20]. Human-induced pluripotent stem cells-EVs promoted cell proliferation, decreased cellular ROS levels and production of γ-H2A.X and SASP, and thus alleviated senescence [21]. Besides, EVs are members of SASP and regulators of human skin homeostasis during senescence [19]. EVs-treated skin fibroblasts showed enhanced proliferation and migration, extracellular matrix protein and growth factors secretions [22]. Similarly, pretreatment with human induced pluripotent stem cells-EVs inhibited the damages of human skin fibroblasts and overexpression of MMP-1/3 [23].

Furthermore, we verified that ED-EVs promoted wound healing in diabetic mice, increased microvascular density, collagen deposition, type II macrophages (CD163^+^)/type I macrophages (CD86^+^) and positive rates of CD31, α-SMA and CD34, and accelerated EMT. Similarly, macrophage-derived EVs or human endothelial progenitor cells-derived EVs inhibited chronic inflammation, enhanced levels of angiogenesis-related proteins, collagen deposition and re-epithelialization, and accelerated wound healing in type 1 diabetic rats [24, 25]. Specific aspects of wound healing include re-epithelialization, neovascularization, blood vessel maturation and increased collagen deposition [10]. Coculture of CD163-overexpressing macrophages and fibroblasts effectively induced re-epithelialization and promoted wound healing [26]. In the process of angiogenesis, CD34-positive cells and α-SMA-positive pericytes are very critical for the healing of skin injury [27]. And mature endothelial cells promote itself differentiation to CD34^+^ cells [28]. Consistently, amniotic ED-EVs promote fibroblast proliferation and migration, thus accelerating wound healing with well-organized collagen fibers and inhibiting scar formation [29].

Moreover, YAP was dephosphorylated and transported to the nucleus after plasma ED-EVs treatment. YAP upregulation is related to blocked cell proliferation and boosted senescence [30]. Restoration of YAP nuclear translocation could rescue skin fibroblast proliferation in Ezrin knockdown cells [31]. YAP is involved in skin repair and re-epithelialization [32]. Platelet-rich plasma-EVs induced proliferation and migration of fibroblasts to improve angiogenesis and re-epithelialization in diabetic wounds via activation of YAP [33]. The PI3K/Akt/mTOR pathway is responsible for the premature senescence of endothelial cells exposed to chronic radiation [16]. Activation of PI3K/Akt/mTOR pathway induced intracellular lipid accumulation by boosting fatty acid synthesis, thus elevating fatty acid oxidation and cell senescence [34]. The activated PI3K/Akt/mTOR pathway not only upregulates the expression of VEGF, FGF and EGF, but also promotes cell growth and migration, angiogenesis and collagen synthesis, induces EMT and stimulates wound healing [35, 36]. EGF and VEGF-C may be involved in scar formation, and stimulate the process of skin wound repair [37]. In the wounds in STZ-induced diabetic mice, suppressed levels of inflammation-related cytokines, tumor necrosis factor-α (TNF-α), IL-8, and IL-6 were achieved after the treatment of an anti-inflammation drug [38]. Interestingly, elevated levels of EGF, FGF, TGF-β, VEGF and PI3K, and decreased levels of proinflammatory cytokines TNF-α and NF-κB p65 were observed in diabetic wound treated with topical mangiferin, which has potential to be used as an agent to promote wound healing in diabetic condition [39]. Of particular note, the PI3K/Akt pathway is implicated in EVs-induced angiogenesis- and proliferation-promoting effects, as well acceleration of diabetic wound healing [33]. Our results also revealed that YAP inhibition or LY294002 (a PI3K/Akt pathway inhibitor) treatment reversed the effect of plasma ED-EVs on fibroblast proliferation.

In conclusion, a significant amount of compelling evidence in the current study supports the function of ED-EVs as a novel therapeutic tool for diabetic wound healing. We summarized that plasma ED-EVs could prevent fibroblast senescence and accelerate skin wound healing in diabetic mice by promoting YAP nuclear translocation and activating the PI3K/Akt/mTOR pathway. However, angiogenesis-related factors EGF and VEGF and pro-inflammatory factors IL-6 and TNF-α in wound healing were not evaluated in our study. In the future, we will make a comprehensive study involving the angiogenesis and inflammation to delve into the mechanism of wound healing. Much more work is needed to understand the differences during all stages of wound healing that occur in the diabetic state, with the goal of finding EV-based therapies to help drive these non-healing wounds to a state of health and integrity.

## MATERIALS AND METHODS

### Ethics statement

This study was approved and supervised by the animal ethics committee of the Second Hospital of Hebei Medical University and conducted in compliance with the Helsinki declaration. All procedures were strictly conducted as per the Code of Ethics. All participants were informed and signed the informed consent. The experiments involving animals were performed with the approval of the Laboratory Animals and Care Committee (IACUC). Significant efforts were made to minimize the number of animals and their suffering.

### Extraction and identification of plasma ED-EVs

Platelet-poor plasma was prepared using 6 mL venous blood obtained from healthy volunteers and stored in 0.50-mL aliquots for ED-EVs enrichment as described [11]. Aliquots of 0.50 mL diluted poor plasma were cultured with 0.15 mL thromboplastin D (Thermo Fisher Scientific, Waltham, MA, USA), followed by adding 0.35 mL Dulbecco’s balanced salt solution, containing protease inhibitor cocktail (Roche Applied Sciences, Indianapolis, IN, USA) and phosphatase inhibitor cocktail (Thermo Fisher Scientific). Following centrifugation, the supernatant was incubated with ExoQuick EV precipitation solution (System Biosciences, Mountain View, CA, USA). Each pellet was resuspended in distilled water with inhibitor cocktails. Two biotinylated monoclonal antibodies against CD31 (clone MEM-05; Thermo Fisher Scientific) and CD146 (Novus biology, Littleton, CO, USA) were used for immunoprecipitation for enrichment of plasma ED-EVs.

Nanosight NS500 system (Malvern Panalytical Co., Ltd, Malvern, Worcestershire, UK) with a G532 nm laser module and a NTA3.1 nanoparticle tracking software was applied to determine the mean diameter (nm) and concentration (particles/mL) of EVs in each suspension. The morphology of enriched ED-EVs was examined by transmission electron microscopy.

CD63, CD81, tumor susceptibility gene 101 (TSG101), vascular cell adhesion molecule 1 (VCAM-1), glycoprotein VI (GPVI) proteins were detected by western blot analysis, with the supernatant-negative control (S-NC) after immunoprecipitation used as the control. The information about the antibodies are shown in Table 1.

**Table 1:** Antibodies used in Western blot analysis.

**Antibody**	**Dilution**	**Cat. No**	**Supplier**
CD63	1:1000	ab59479	Abcam
CD81	1:1000	ab79559	Abcam
TSG101	1:1000	ab30871	Abcam
VCAM-1	1:1000	#13662	CST
GPVI	1:10000	ab129019	Abcam
H2A.X	1:5000	ab11175	Abcam
p16^INK4A^	1:2000	ab108349	Abcam
YAP1	1:5000	ab52771	Abcam
p-YAP1	1:10000	ab76252	Abcam
CTGF	1:1000	ab6992	Abcam
VEGF	1:1000	ab32152	Abcam
bFGF	1:1000	ab92337	Abcam
AKT1	1:1000	ab235958	Abcam
p-AKT1	1:5000	ab81283	Abcam
mTOR	1:2000	ab2732	Abcam
p-mTOR	1:1000	ab109268	Abcam
Histone H3	1:1000	ab176842	Abcam
β-actin	1:1000	ab8277	Abcam

To verify the effect of ED-EVs, we prepared ED-EVs and S-NC with the same total protein content. The protein content of S-NC and ED-EVs was measured using a bicinchoninic acid (BCA) kit (Thermo Fisher Scientific) and then standardized. In the experiment, 5 μg/mL and 50 μg/mL protein concentration was used.

### Enzyme-linked immunosorbent assay (ELISA)

After EV cleavage, ED-EVs cargo proteins were quantified by ELISA kits for VCAM-1, GPVI, and tetraspanning exosome marker CD81 (Cusabio; American Research Products, Waltham, MA, USA). CD81 served as a surrogate marker of EV concentration against which each protein was normalized.

The levels of senescence-associated secretory phenotype (SASP)-related proteins matrix metalloproteinase (MMP)-3, interleukin (IL)-6 and IL-1β in human skin fibroblasts and mouse skin homogenates were detected as per the corresponding instructions of an MMP-3 Human ELISA kit (Thermo Fisher Scientific), IL-6 Human ELISA kit (Thermo Fisher Scientific) and Human IL-1β Quantikine ELISA kit (DLB50, R&D Systems, Minneapolis, MN, USA).

### Culture of human skin fibroblasts

Human skin fibroblasts were obtained from skin tissues of type 2 diabetic patients and healthy volunteers in the Second Hospital of Hebei Medical University. Human skin samples were cut into small pieces and cultured overnight in 1 mg/mL type I collagenase. The detached dermal mixture was filtered via a 70-μm cell filter and cultured in Dulbecco's modified Eagle's medium (DMEM) containing 10% fetal bovine serum (FBS). The 3-6 generations of cells were used for subsequent experiments. The cells were identified as fibroblasts by immunocytochemistry. The antibodies are listed in Table 2. The skin fibroblasts obtained from healthy volunteers were named as HSF while that from diabetic patients were named as DSF.

**Table 2:** Antibodies used in immunocytochemistry.

**Antibody**	**Dilution**	**Cat. No**	**Supplier**
collagen I	1:100	ab34710	Abcam
α-SMA	1:500	ab32575	Abcam
IgG	1:2000	ab205718	Abcam
Vimentin	1:100	ab8978	Abcam

Human epidermal cell line HaCaT (American Type Culture Collection (Manassas, Virginia, USA) was cultured in DMEM with 10% FBS, 2 mM/L glutamine, 10 ng/mL epidermal growth factor (EGF) (Sigma-Aldrich, Merck KGaA, Darmstadt, Germany) and 1 μg/mL hydrocortisone (Sigma-Aldrich). Both HaCaT cells and fibroblasts were maintained in saturated moist air (containing 5% CO_2_) at 37°C.

### Cell transfection and grouping

The Yes-associated protein (YAP) cDNA was cloned and the overexpression vector pcDNA3.1-YAP was constructed. The short-interfering (si)-YAP-1, si-YAP-2, si-NC (Thermo Fisher Scientific) were designed and synthesized. The siRNA and overexpression vectors were respectively transfected into fibroblasts using Lipofectamine 2000 (Invitrogen Inc., Carlsbad, CA, USA) according to the instructions.

Cells were assigned into blank group (untreated HaCaT cells or fibroblasts), S-NC group (HaCaT cells or fibroblasts were incubated with 5 μg/mL or 50 μg/mL S-NC), ED-EVs group (HaCaT cells or fibroblasts were incubated with 5 μg/mL or 50 μg/mL ED-EVs), ED-EVs + si-NC group (HaCaT cells or fibroblasts in the ED-EVs group were further transfected with si-NC), ED-EVs + si-YAP group (HaCaT cells or fibroblasts in the ED-EVs group were further transfected with si-YAP-1 or si-YAP-2), NC group (HaCaT cells or fibroblasts were transfected with pcDNA3.1 empty vector), YAP group (HaCaT cells or fibroblasts were transfected with pcDNA3.1-YAP overexpression vector), ED-EVs + TFA group (HaCaT cells or fibroblasts in the ED-EVs group were further treated with YAP inhibitor Super-TDU TFA), ED-EVs + LY294002 group (HaCaT cells or fibroblasts in the ED-EVs group were further treated with PI3K/Akt/mTOR inhibitor LY294002). YAP inhibitor Super-TDU TFA and PI3K/Akt/mTOR inhibitor LY294002 were purchased from MedChemExpress (Monmouth Junction, NJ, USA).

### Senescence-associated-β-galactosidase (SA-β-Gal) assay

Cells were washed in PBS, fixed in 4% paraformaldehyde for 10 minutes, washed, and incubated with fresh SA-β-Gal stain solution (pH 6.0), including potassium ferricyanide (5 mM), potassium ferrocyanide (5 mM), sodium dihydrogen phosphate (0.4 mM), sodium hydrogen phosphate (92 mM), sodium chloride (150 mM), magnesium dichloride (2 mM) and 5-bromo-4-chloro-3-indolyl-β-d-galactopyranoside (1 mg/mL) at 37°C with CO_2_. Staining was evident in 2-4 hours and maximal in 12-16 hours. SA-β-Gal assay and Fast Red staining were carried out after the skin tissues were frozen and sectioned at 7-8 μm.

### Preparation of nuclear and cytoplasmic extracts

An NE-PER nuclear and cytoplasmic extraction kit (Pierce Biotechnology Inc, Rockford, IL, USA) was used to prepare nuclear and cytoplasmic extracts from cultured cells step by step. The protein concentration was determined using a BCA kit. Then the protein level of the extracts was determined by western blot analysis.

### Western blot analysis

The treated cells were lysed for 30 minutes in cold radio-immunoprecipitation assay containing protease inhibitor cocktail. Then, the lysate was centrifuged at 4°C for 20 minutes at 16000 g, and the supernatant was harvested. The protein concentration was measured using a BCA kit. Equal amount of proteins (20 μL/well) were loaded into wells for sodium dodecyl sulfate polyacrylamide gel electrophoresis. Subsequently, the proteins were transferred to polyvinylidene fluoride membranes. After that, the membranes were blocked with 5% skimmed milk powder for 1 hour and probed with primary antibodies (Table 1) at 4°C overnight. After that, the membranes were incubated with secondary antibody IgG (1:2000, ab205718, Abcam). Image J (National Institutes of Health, Bethesda, Maryland, USA) was applied for gray value analysis with β-actin as a reference.

### Detection of intracellular reactive oxygen species (ROS) level

ROS level in cells was detected using the H2DCFDA probe (MedChemExpress). Cells were treated with S-NC or ED-EVs, and then incubated with 10 μm H2DCFDA for 1 hour. Afterwards, the fluorescence intensity was observed under a fluorescence microscope (Olympus). The fluorescence intensity was analyzed by Image J software.

### Determination of cell proliferation

Cell proliferation was detected using cell counting kit-8 (CCK-8) assay and 5-Ethynyl-2’-Deoxyuridine (EdU) labeling assay. According to the CCK-8 instructions (HY-K0301, MedChemExpress), cells were cultured at 5000 cells/well in 96-well plates with 5 μg/mL or 50 μg/mL ED-EVs at 37°C. S-NC with the same volume of proteins was served as the control, and plates without cells were served as the blank. On day 0, 3, 5 and 7, 20 μL CCK-8 solution and 180 μL fresh media were loaded to each well at each time point, and incubated at 37°C for 1 hour. The absorbance at 450 nm was measured using a spectrophotometer. Cell survival/proliferation = the optical density (OD) of tested wells - the absorbance of blank wells.

According to the instructions of EdU kit (Invitrogen), 10 μm EdU was given to cells in each well. After 24 hours, the cells were fixed and cleared, and treated with reaction mixture and 4’,6-diamidino-2-phenylindole (DAPI), following observation under a fluorescence microscope (Olympus).

### Transwell assay

HaCaT cells or skin fibroblasts in each group were collected, treated with serum-free medium (containing 0.1% bovine serum albumin) and resuspended to 1 × 10^5^ cells/mL, and then placed in the apical chambers. DMEM containing 10% FBS was filled in the basolateral chambers. Transwell chambers were cultivated at 37°C with 5% CO_2_ for 24 hours. Then the filter membrane was taken out, washed in PBS, and fixed with 0.5% glutaraldehyde. After that, cells were stained with crystal violet solution at 37°C with 5% CO_2_ for 24 hours. Under the microscope, 5 fields (200 ×) were randomly selected for cell counting.

### Skin-wound model in diabetic mice

All mice were purchased from the Shanghai branch of Beijing Vital River Laboratory Animal Technology Co., Ltd. (Shanghai, China) (SYXK (Shanghai) 2017-0015). The 4-week-old mice were fed with high-fat and high-glucose diet for 4 weeks after adapting to the environment for 1 week. All animals were fasted 12 hours before modeling. Fresh streptozocin (STZ) solution (2% STZ, prepared from 0.1 moL/L citric acid sodium citrate buffer, pH = 4.4) was injected into each mouse by intraperitoneal injection at 50 mg/kg. After 72 hours, blood glucose concentration in tail vein blood of mice was measured. Mice with blood glucose concentration > 16.7 mmoL/L were regarded as successful diabetic models.

One day before the operation, the diabetic mice were taken for skin preparation on the back. After inhaling pentobarbital for anesthesia, mice were placed in a prone position and their limbs were fixed. In the middle of the back, a circular operation area (1 × 1 cm^2^) was marked with methylene blue. A full-thickness skin defect was made by scalpel and eye scissors, and the gauze was used to stop bleeding.

Mice were assigned into control group (n = 48, injected with 100 μL PBS every 3 days within 14 days after skin wound), S-NC group (n =48, injected with 100 μL 50 μg/mL S-NC every 3 days within 14 days after skin wound), and ED-EVs group (n =48, injected with 100 μL 50 μg/mL ED-EVs every 3 days within 14 days after skin wound). Six mice in each group were used for hematoxylin and eosin (HE) staining, 18 for immunofluorescence, 15 for western blot analysis, 3 for Masson staining, and 6 for wound healing analysis.

### Wound healing analysis

The wound diameter at 0, 3, 7, 10, and 14 days after skin wound was measured. Wound closure was assessed as a percentage of the reduction in wound area. When the wound area was gradually reduced, the wound margin was traced on a transparent paper, and then the tracing was placed on the square paper and the square number was calculated. Wound closure was expressed as a percentage of the reduction of the area of original wound, and was calculated. The wound closure percentage on day n = (area on day 0 - area on day n)/area on day 0 × 100, and the area on day 0 was defined by the area of original wound obtained immediately after skin wound.

### Histological examination

On the 3^rd^, 7^th^ and 14^th^ day after skin wound, mice were euthanized by intraperitoneal injection of pentobarbital injection (800 mg/kg) [40, 41]. Skin tissue samples including the injured tissues and surrounding normal skin tissues were then collected. The collected tissues were immediately fixed in 10% neutral buffered formalin, embedded in paraffin and then sectioned. HE staining was employed to observe tissue morphology on the 3^rd^ and 14^th^ day, Masson staining was utilized to assess collagen deposition on the 14^th^ day, and immunofluorescence was applied for detection of protein expression on the 3^rd^, 7^th^ and 14^th^ day.

After hydration, sections were sealed with 1.5% goat serum and incubated overnight with primary antibodies, and then incubated with secondary antibody for 1 hour. After PBS washing, DAPI was applied for nuclear staining. The images were examined under the confocal laser scanning microscope (Carl Zeiss, Oberkochen, Germany). The antibodies used in immunofluorescence are listed in Table 3.

**Table 3:** Antibodies used in immunofluorescence.

**Antibody**	**Dilution**	**Cat. No**	**Supplier**
CD31	1:100	ab215911	Abcam
CD34	1:100	ab81289	Abcam
cytokeratin 14	1:100	ab77684	Abcam
cytokeratin 10	1:50	ab194231	Abcam
CD163	1:200	ab182422	Abcam
CD86	1:200	ab218757	Abcam
vimentin	1:100	ab194719	Abcam
IgG	1:2000	ab205718	Abcam

### Statistical analysis

Statistical analysis was conducted by SPSS21.0 (IBM Corp. Armonk, NY, USA). The Kolmogorov-Smirnov test was used to check whether the data were normally distributed. The measurement data were exhibited in mean ± standard deviation. The *t* test was applied for comparisons between two groups, while one-way or two-way analysis of variance (ANOVA) and Tukey’s multiple comparisons test were applied for multiple groups. The *p* value was calculated using a two-tailed test and *p* < 0.05 meant a statistical difference.
